# Temperature Sensing of Deep Abdominal Region in Mice by Using Over-1000 nm Near-Infrared Luminescence of Rare-Earth-Doped NaYF_4_ Nanothermometer

**DOI:** 10.1038/s41598-018-35354-y

**Published:** 2018-11-19

**Authors:** Shota Sekiyama, Masakazu Umezawa, Shuhei Kuraoka, Takuji Ube, Masao Kamimura, Kohei Soga

**Affiliations:** 1 Department of Materials Science and Technology, Faculty of Industrial Science and Technology, Tokyo University of Science, 6-3-1 Niijuku, Katsushika-ku, Tokyo 125-8585 Japan; 2 Imaging Frontier Center (IFC), Research Institute for Science and Technology (RIST), Tokyo University of Science, 2641 Yamazaki, Noda, Chiba 278-8510 Japan

## Abstract

Luminescence nanothermometry has attracted much attention as a non-contact thermal sensing technique. However, it is not widely explored for *in vivo* applications owing to the low transparency of tissues for the light to be used. In this study, we performed biological temperature sensing in deep tissues using β-NaYF_4_ nanoparticles co-doped with Yb^3+^, Ho^3+^, and Er^3+^ (NaYF_4_: Yb^3+^, Ho^3+^, Er^3+^ NPs), which displayed two emission peaks at 1150 nm (Ho^3+^) and 1550 nm (Er^3+^) in the >1000 nm near-infrared wavelength region, where the scattering and absorption of light by biological tissues are at the minimum. The change in the luminescence intensity ratio of the emission peaks of Ho^3+^ and Er^3+^ (*I*_Ho_/*I*_Er_) in the NaYF_4_: Yb^3+^, Ho^3+^, Er^3+^ nanothermometer differs corresponding to the thickness of the tissue. Therefore, the relationship between *I*_Ho_/*I*_Er_ ratio and temperature needs to be calibrated by the depth of the nanothermometer. The temperature-dependent change in the *I*_Ho_/*I*_Er_ was evident at the peritoneal cavity level, which is deeper than the subcutaneous tissue level. The designed experimental system for temperature imaging will open the window to novel luminescent nanothermometers for *in vivo* deep tissue temperature sensing.

## Introduction

Nanosized materials have become popular in the field of nanomedicine as they provide new perspectives on imaging, diagnosis, and therapy. Nanotechnology has offered investigators novel materials and tools against challenging problems that have remained unresolved for decades. Indeed, nanotechnology has allowed *in vivo* deep tissue imaging^1–3^, single photon emission tomography (SPECT)/computed tomography (CT) *in vivo* dual-modal imaging^4^, safe and controlled drug delivery^5^, and near-infrared (NIR)-induced photodynamic therapy^6^. In addition, nanotechnology has also enabled thermal therapy techniques such as photothermal therapy (PTT)^7–9^, making preeminent contributions to clinical studies. The application of nanotechnology to thermal therapy, in particular, has attracted much attention because the PTT procedure allows the destruction of cancer cells by heating them at the physiological temperature using external light irradiation, which is critical for achieving ideal results. In addition, it is necessary to develop methods for measuring the temperature in deep tissues because temperature is one of the most crucial parameters for controlling biological events^10^.

Recently, luminescence nanothermometry (LNTh) has been developed in biochemical research. LNTh is a non-contact thermal sensing technique that uses luminescent nanoparticles (NPs) in the physiological temperature range (20–50 °C)^11–13^. This thermal sensing helps us to study cellular processes such as gene expression, metabolism, or cell division, as they are usually accompanied by a change in the temperature. For example, the existence of a temperature gradient within cells was revealed using intracellular temperature mapping^14^. LNTh is suitable for achieving a full understanding of the cellular processes. Thus, it is desirable that LNTh is applied in *in vivo* experiments.

Although LNTh has been used in different *in vitro* experiments, it has not been widely explored for *in vivo* deep tissues. Indeed, although it has been partially applied in a living organism, there is a serious problem in terms of light transparency. Light penetration of tissues is a key factor for achieving bioimaging corresponding to the observation depth, which is determined by the wavelength of light. The ultraviolet (UV)/visible (VIS) wavelength range, for example, has been used for fluorescence imaging despite high absorption and scattering of these wavelengths by biological tissues. Most luminescent thermometers have their excitation and emission wavelengths in the visible range, where the depth of light penetration is limited to <1 mm^15^. These VIS-excited/VIS-emitting probes were used for small semitransparent organisms such as fly larvae^16^ and the nematode, *Caenorhabditis elegans*^17^_,_ owing to low penetration of light. *In vivo* use of LNTh is limited to small semitransparent organisms because of the low light transparency of living tissues to VIS light. Therefore, a wavelength range suitable for deep tissue imaging is required.

To overcome this problem, NIR wavelength region has been focused for *in vivo* applications. This wavelength domain, the so-called “biological window,” is well known to be less scattered and absorbed by biological tissues, and is thus utilized for fluorescence imaging in deep tissues^18,19^. This domain is distinguished according to the wavelength range as the first (NIR-I: 650–950 nm), second (NIR-II: 1000–1350 nm), and third (NIR-III: 1500–1800 nm) biological window. Light in each wavelength region plays a different role in deep tissue bioimaging^15,18,20^. The NIR-I wavelength region may not be suitable for deep tissue imaging owing to the autofluorescence of the tissues; however, it is commonly used as an effective NIR laser excitation source because of its extraordinary tissue penetration^21,22^. Light penetration depth could be expanded to ≈1 cm in the NIR-I wavelength region, while it is limited to <1 mm in the wavelength range of UV/VIS light^15^. The NIR-II wavelength region is appropriate for NIR imaging because of its relatively low absorption by water and fat. The NIR-III wavelength region also provides high spatial resolution with minimized scattering coefficient of tissue^15,23–27^. Moreover, the NIR light in both NIR-II and NIR-III wavelength regions has been explored for high-resolution *in vivo* deep imaging because of the availability of commercial fluorescence cameras based on InGaAs detectors^18^. In over-1000 nm (OTN-) NIR wavelength domain including NIR-II and NIR-III, in particular, deep tissue imaging could be accomplished with InGaAs detectors because of the low autofluorescence, low scattering, and low absorption. Thus, OTN-NIR fluorescence imaging would allow the measurement of temperature in deep tissues.

In the OTN-NIR wavelength region, possibilities of *in vivo* applications using luminescent NPs have therefore been opened^28^. Indeed, there are various nanothermometers capable of recording subcutaneous tissue (at ≈1-mm depth) temperatures in this wavelength region. For example, del Rosal *et al*. reported infrared-emitting QDs for thermal therapy with real-time subcutaneous temperature feedback^29^. Ruiz *et al*., showed Ag/Ag_2_S nanocrystals for high-resolution subtissue (1-mm depth) thermal sensing^30^. Ximendes *et al*. also reported core-shell nanothermometers that can measure *in vivo* subcutaneous temperature^31,32^. These nanothermometers capable of measuring subcutaneous temperature give us an understanding of the temperature change during thermal therapy. However, temperature sensing in deeper parts, such as the peritoneal cavity, have not accomplished with these luminescent nanothermometers. Thus, it is imperative to design luminescent nanothermometers capable of measuring the temperature in the deeper tissue.

Our group has developed and reported the synthesis of a β-NaYF_4_: Yb^3+^, Ho^3+^, Er^3+^ nanothermometer for ratiometric LNTh in the OTN-NIR region^33^. The sensing is attributed to the change in the luminescence intensity ratio (LIR) of the Ho^3+^ emission (*I*_Ho_) at 1150 nm to that of Er^3+^ emission (*I*_Er_) at 1550 nm as a function of temperature under 980-nm laser excitation. This ratiometric temperature sensing is not affected by the concentration of the probe, thus reducing the dependency of sensing on measurement conditions. Thus, the LIR approach would allow for accurate temperature sensing. The use of the nanothermometer in our previous study was limited to *in vitro* experiments, and its application for experiments is required to determine the temperature in deeper parts of the body by the LIR approach.

In this work, we performed temperature sensing in deeper tissues at the peritoneal cavity level instead of the subcutaneous tissue level using a rare-earth based nanothermometer that shows temperature-dependent luminescence in the NIR-II and NIR-III wavelength regions. The LNTh is based on a tri-doped NaYF_4_ nanocrystal consisting of luminescent Yb^3+^, Ho^3+^, Er^3+^ ions, which can be used as a ratiometric thermal sensor using the ratio of the *I*_Ho_ at 1150 nm and *I*_Er_ at 1550 nm under 980-nm laser excitation to determine the accurate temperature of deeper tissues such as the peritoneal cavity. Preliminary investigation of the temperature-dependent LIR using biological tissues *in vitro* suggested the necessity of obtaining a depth-corresponded calibration curve to be applied for temperature sensing in deep tissues. The ability of the nanothermometer to measure temperature in deeper parts of the body such as the peritoneal cavity was also investigated.

## Results

### Characterization of NaYF_4_: Yb^3+^, Ho^3+^, Er^3+^ NPs

**Figure 1:**
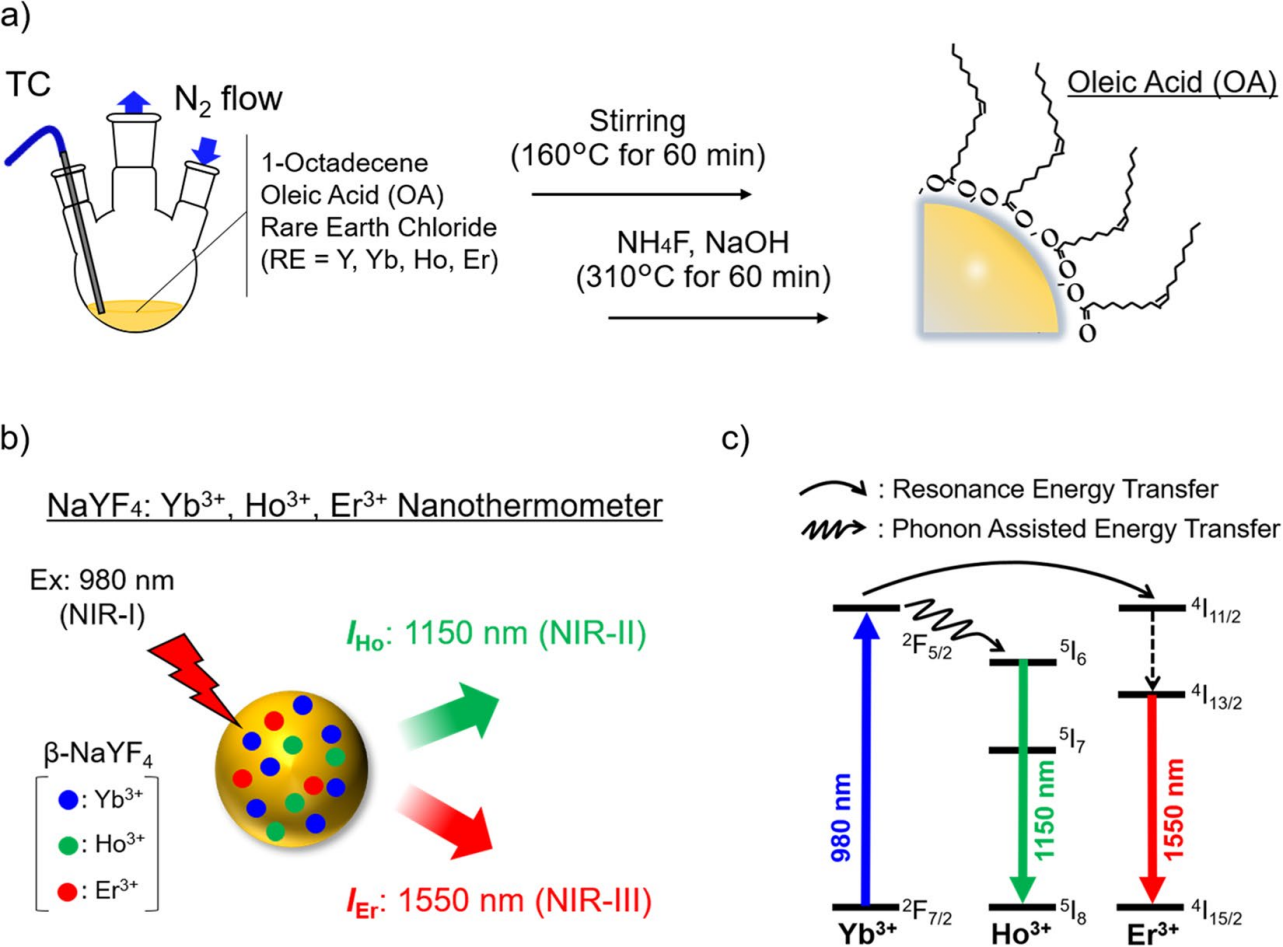
Schematics showing the fabrication and emission of NaYF_4_ NPs serving as the nanothermometer^31^. (**a**) Schematic of the preparation of NaYF_4_: Yb^3+^, Ho^3+^, Er^3+^ NPs. The oleic acid-capped NaYF_4_:Yb^3+^, Ho^3+^, Er^3+^ NPs were synthesized by a thermal decomposition method and the NPs were finally dispersed in cyclohexane. (**b**) Schematic illustration of the NaYF_4_ NPs emitting at 1150 nm (NIR-II) and 1550 nm (NIR-III) under 980-nm (NIR-I) laser excitation. (**c**) Simplified energy scheme of the Yb^3+^, Ho^3+^, and Er^3+^ emitting centers representing the excitation, resonance energy transfer, and phonon-assisted energy transfer. Under 980-nm excitation (blue line), they present the emission bands ascribed to Ho^3+^ (1150 nm, green line) and Er^3+^ (1550 nm, red line).

The NaYF_4_: Yb^3+^, Ho^3+^, Er^3+^ NPs were synthesized by the thermal decomposition method, as shown in Fig. 1. After several trials^34^, the Yb^3+^, Ho^3+^, and Er^3+^ concentrations were respectively fixed at 20, 3, and 0.5 mol%. The NP surface was capped by oleic acid (OA) and they were dispersed in cyclohexane. The designed NPs show OTN-NIR emission bands under 980-nm excitation owing to Yb^3+^ ion used as a sensitizer to actuate both Ho^3+^ and Er^3+^ ions.

**Figure 2:**
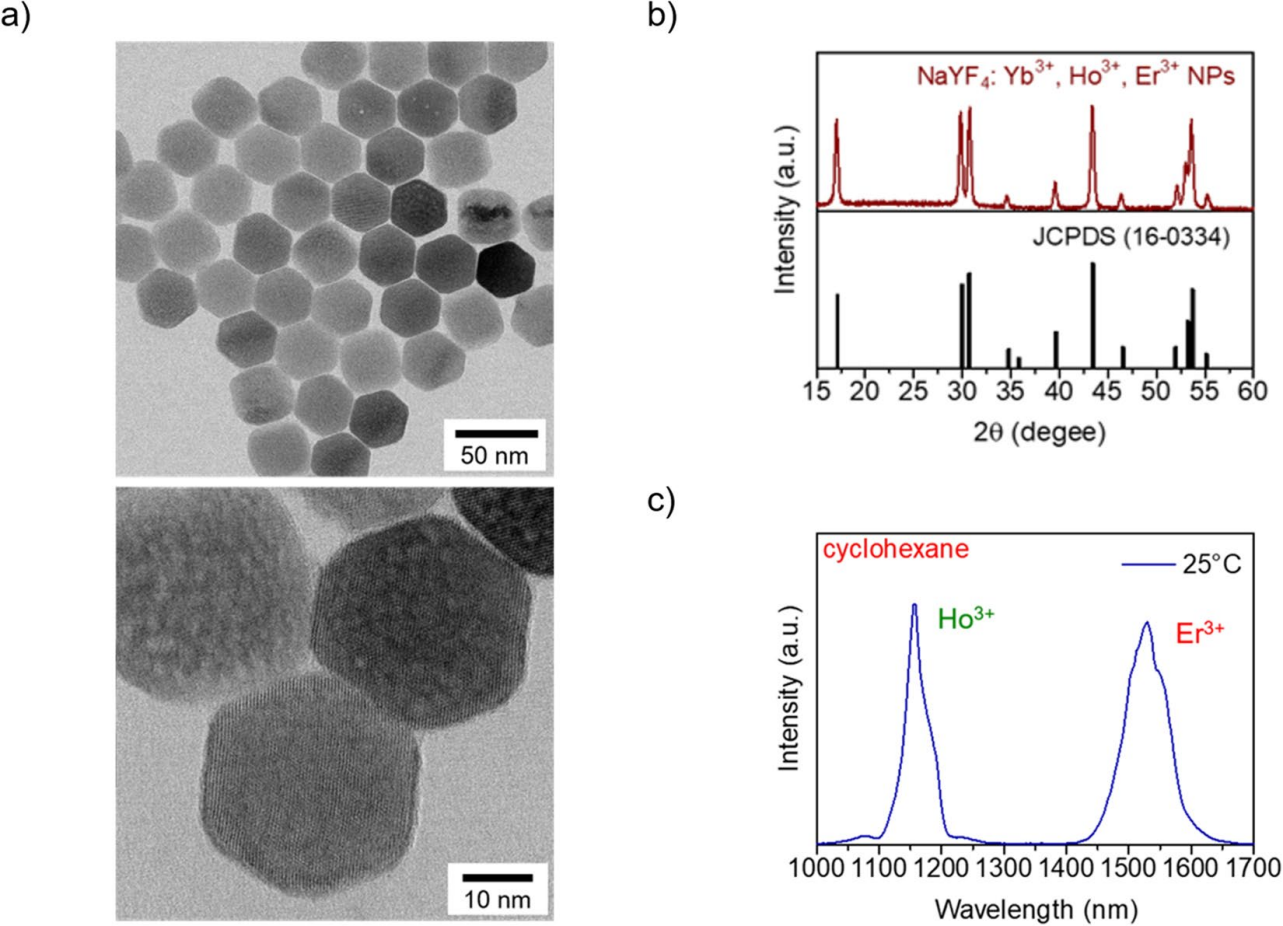
Characterization of the NaYF_4_ NPs. (**a**) Representative TEM image of the NaYF_4_ NPs. (**b**) XRD pattern of the synthesized NaYF_4_ NPs (top panel); the reference XRD pattern of β-NaYF_4_ (JCPDS: 16-0334) is shown in the bottom panel. (**c**) Room-temperature OTN-NIR emission spectrum of the NaYF_4_ NPs in cyclohexane at a concentration of 11 mg/mL under 980-nm laser excitation. The laser power was 4.22 W.

The average NPs diameter and crystal phase of the NPs were respectively evaluated via transmission electron microscopy (TEM) and X-ray diffraction. Figure 2a shows a representative TEM image of OA-capped NaYF_4_: Yb^3+^, Ho^3+^, Er^3+^ (NaYF_4_) NPs. The TEM image shows hexagonal particles with an average size of 35 ± 2 nm. The NaYF_4_ NPs are crystalline with pure β-phase; all the peaks could be indexed to JCPDS data (16-0334) (Fig. 2b). The high colloidal stability of this sample is apparent by the hydrodynamic diameter determined by dynamic light scattering in cyclohexane (see Fig. S1, Supplementary information). The room-temperature emission spectrum of the NaYF_4_ NPs dispersed in cyclohexane under NIR 980-nm laser excitation (Fig. 2c) shows emission bands of Ho^3+^ and Er^3+^ centered at 1150 and 1550 nm, respectively.

### Thermal Sensitivity of the NaYF_4_ NPs

**Figure 3:**
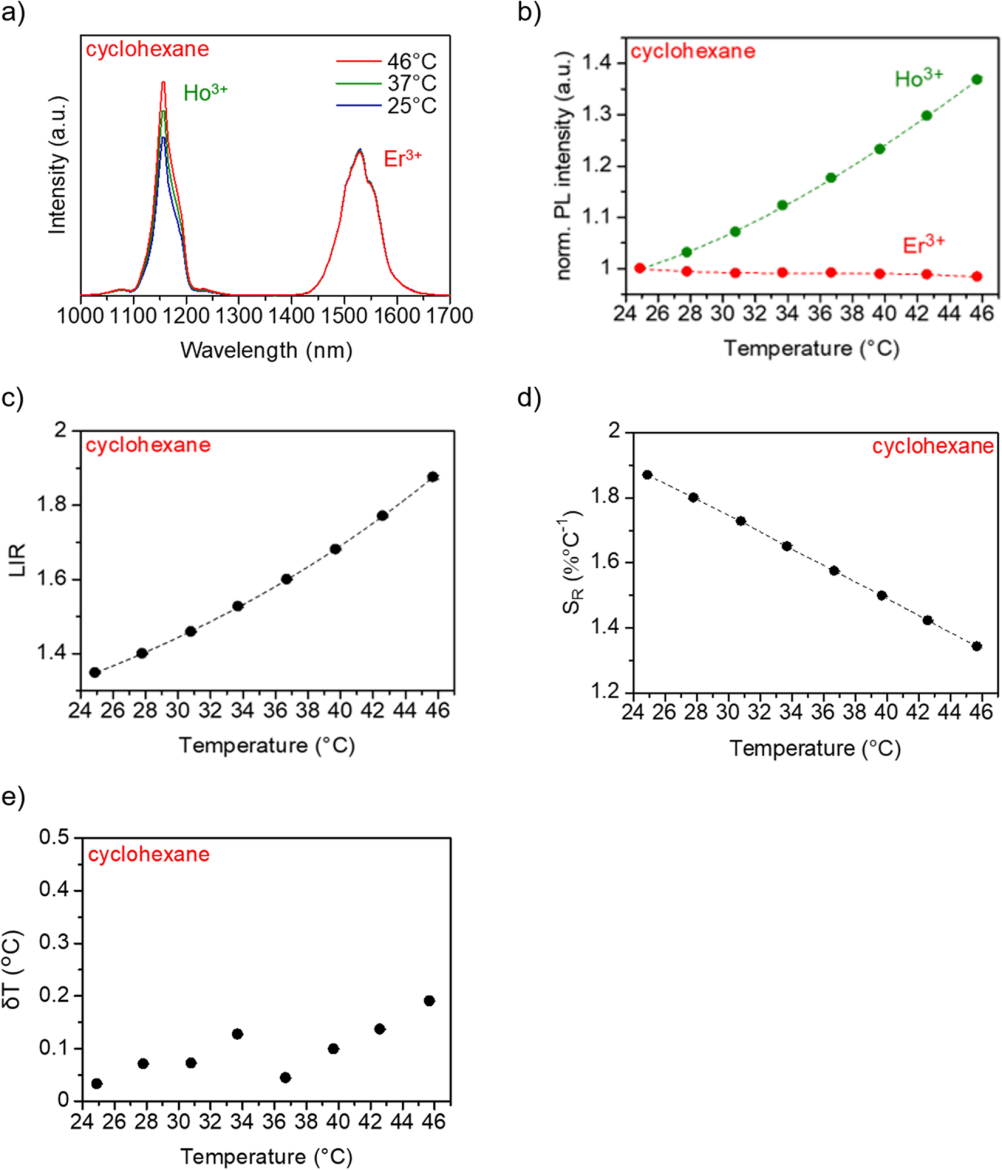
Optical characterization of the NaYF_4_ NPs. (**a**) OTN-NIR emission spectra of NaYF_4_ NPs obtained at 25, 37, and 46 °C under 980-nm excitation. (**b**) Normalized PL intensity of Ho^3+^ and Er^3+^. (**c**) Calibration curve of the NaYF_4_ NPs of the relationship between *I*_Ho_/*I*_Er_ ratio and temperature. (**d**) Relative thermal sensitivity and (**e**) temperature uncertainty as functions of temperature for the NaYF_4_ NPs. The NPs were dispersed in cyclohexane at a concentration of 11 mg/mL. The laser power was 4.22 W.

The capability of the NaYF_4_ NPs for LNTh in the NIR-II and NIR-III wavelength regions was verified by analyzing their emission spectra under 980-nm excitation in the physiological (25−46 °C) temperature range. Figure 3 includes the emission spectra, normalized photoluminescence (PL) intensity, LIR, that is, the ratio of the integrated emission peak intensity of Ho^3+^ (*I*_Ho_) and Er^3+^ (*I*_Er_) (*R* = *I*_Ho_/*I*_Er_), and the relative thermal sensitivity of the NaYF_4_ NPs dispersed in cyclohexane. Figure 3a shows the temperature-dependent emission spectra of NaYF_4_ NPs (obtained at 25, 37, and 46 °C) under 980-nm excitation. The temperature-dependent variations in the spectral characteristics indicate the difference in the emission of each rare-earth ion corresponding to temperature change. Indeed, Ho^3+^ emission increased with increasing temperature, whereas the Er^3+^ emission remained almost consistent in the physiological temperature range, as shown in Fig. 3b. In other words, the integrated emission peak intensity of Ho^3+^ (*I*_Ho_) is sensitive to temperature changes, while that of Er^3+^ (*I*_Er_) is unresponsive to temperature changes. From the experimental data shown in Fig. 3c, the relative thermal sensitivity achievable with each of the intensity ratio can be calculated. Here, the relative thermal sensitivity is defined as *S*_R_ = (1/*R*)·(*dR*/*dT*)^11,35^. The results are included in Fig. 3d and can be summarized as follows: at 25 and 46 °C, the NaYF_4_ NPs present relative thermal sensitivities of 1.87 × 10^−2^ °C^−1^ and 1.34 × 10^−2^ °C^−1^, respectively, using *R* as the temperature-sensitive parameter.

In addition, the temperature uncertainty *δT* about *I*_Ho_/*I*_Er_ for our NaYF_4_ NPs is estimated in accordance with the minimal resolvable temperature. *δT* is given by the product of 1/*S*_R_, which is attributed to the intrinsic properties of the material, and the *δR*/*R*, which is the relative uncertainty of the thermometric parameter given by the experimental instrumentation in use^36^. In the present study, the *δR*/*R* is less than 0.26%; thus the *δT* for the NaYF_4_ NPs is less than 0.20 °C in the range of 25−46 °C (Fig. 3e).

### Thermal Sensitivity of the NaYF_4_ NPs in Biological Tissues

After the evaluation of temperature-dependent changes in the emission spectra of NaYF_4_ NPs dispersed in cyclohexane, the emission spectra corresponding to the thickness of the tissue were evaluated using biological tissues under 980-nm laser excitation in the physiological temperature (25−46 °C) range. An experiment for the application of the NaYF_4_ NPs for LNTh in deep tissues was performed. The suitability of NaYF_4_ NPs for LNTh was evaluated by acquiring the emission detected through the biological tissues. The mouse skin and skin/peritoneal muscle were placed on the outer walls of the sample cuvette and the OTN-NIR emission spectra of NaYF_4_ NPs were collected at different temperatures. Even though we cannot hold another tissue specimen additionally at the excitation end in this measurement system, the relationship between *I*_Ho_ and *I*_Er_ will not change by attenuation of the excitation laser with tissues because both will decrease according the laser attenuation in the same manner.

**Figure 4:**
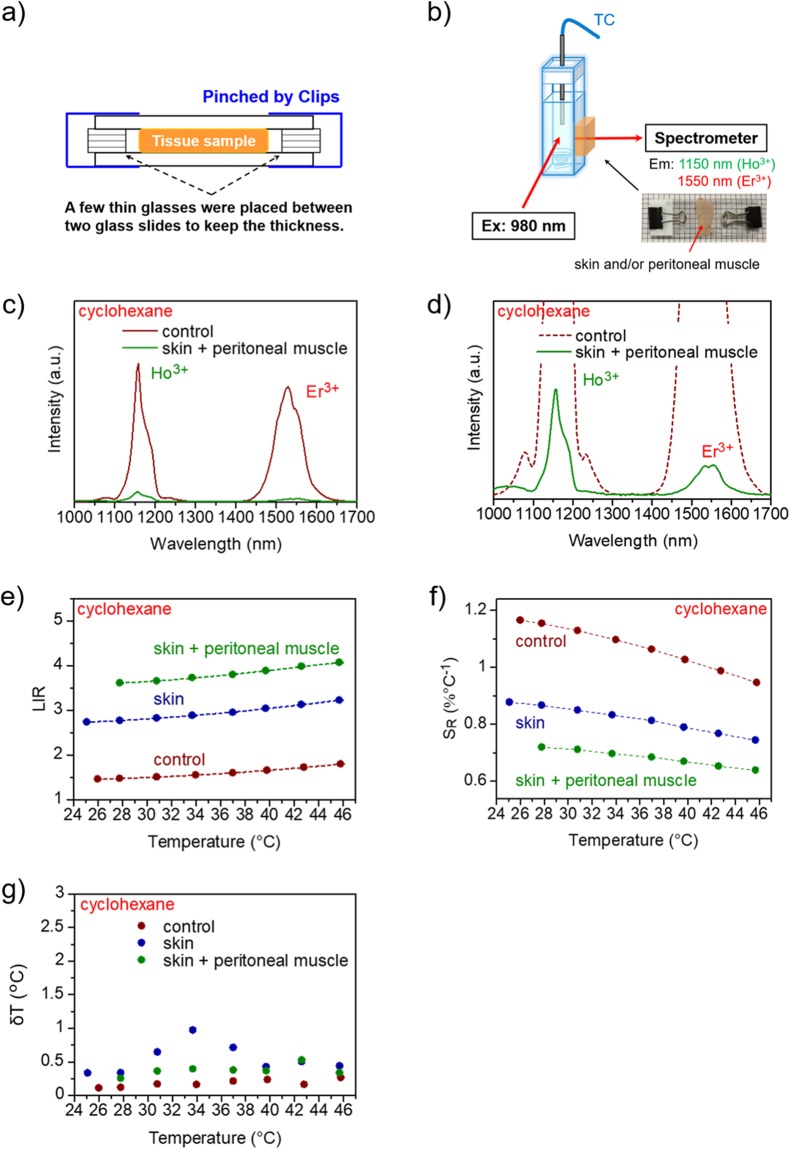
Temperature-dependent OTN-NIR emission spectra of NaYF_4_ NPs recorded using biological tissues (skin and/or peritoneal muscle). (**a**) Schematic representation of the biological tissues used for obtaining the emission spectra. Tissue samples were placed between two glass slides, with the gap maintained at 880 (skin) or 1330 μm (skin + peritoneal muscle). (**b**) Schematic illustration of recording temperature-dependent emission spectra with biological tissues. The tissue samples were placed between the cuvette and detector of the spectrometer. (**c**) Comparison of the luminescence intensity of the NaYF_4_ NPs only (control) vs. that with skin + peritoneal muscle and (**d**) enlarged view of c. (**e**) Comparison of the LIR of the NaYF_4_ NPs (control, skin, and skin + peritoneal muscle). (**f**) Comparison of the relative thermal sensitivity (*S*_R_) and (**g**) the temperature uncertainty for NaYF_4_ NPs using skin and peritoneal muscle. The NPs were dispersed in cyclohexane at a concentration of 30 mg/mL. The laser power was 4.22 W.

Figure 4 includes schematic representations of the biological tissues and recording of the temperature-dependent emission spectra using skin and/or peritoneal muscle and the emission spectra, LIR, and relative thermal sensitivity of NaYF_4_ NPs dispersed in cyclohexane. In our designed system, the biological tissues were placed between two glass slides, wherein a few thin glass slides were planted on either side of the biological tissue to maintain the thickness, as shown in Fig. 4a,b. Figure 4c compares the OTN-NIR luminescence intensity of NaYF_4_ NPs obtained using skin and peritoneal muscle with that obtained without the tissues (control). Figure 4d shows an enlarged view of Fig. 4c, revealing that the Ho^3+^ and Er^3+^ emission intensity of the NaYF_4_ NPs decreases substantially in the presence of tissues as compared with that of the control. OTN-NIR emission of NaYF_4_ NPs could however be detected through the biological tissues under 980-nm laser excitation (Fig. 4d). Figure 4e exhibits three different calibration curves corresponding to the thickness of the tissues; it shows the LIR of NaYF_4_ NPs in the presence of the skin and/or peritoneal muscle (shown as control, skin, and skin + peritoneal muscle) under 980-nm laser excitation in the biological temperature range. The LIR values (Ho^3+^/Er^3+^) at 34 °C are 1.55 (control), 2.88 (skin), and 3.73 (skin + peritoneal muscle), respectively.

Furthermore, Table 1 shows the slopes of the three different calibration curves shown in Fig. 4e (control, skin, and skin + peritoneal muscle). The relationships between the LIR and the temperature are: (control) LIR = 0.0169 T(°C) + 0.995 (R^2^ = 0.988), (skin) LIR = 0.0239 T(°C) + 2.10 (R^2^ = 0.990), and (skin + peritoneal muscle) LIR = 0.0261 T(°C) + 2.86 (R^2^ = 0.994) in the range of 25–46 °C. In particular, each slope is different and the slopes for control, skin, and skin + peritoneal muscle are 0.017, 0.024, and 0.026, respectively. In addition, Fig. 4f shows the relative thermal sensitivity (*S*_R_) of NaYF_4_ NPs in the presence of the biological tissues. The relative thermal sensitivity decreased as the thickness increased. The *S*_R_ is reduced by 22−24% per 1-mm-thick tissue due to the temperature rise of 1 °C in the present study. In the case of thermometry through the biological tissues, the *δR*/*R* is less than 0.8%; thus the temperature uncertainty *δT* about *I*_Ho_/*I*_Er_ for our NaYF_4_ NPs is less than 1.0 °C in the range of 25–46 °C (Fig. 4g).

**Table 1:** Comparison of the slope of the calibration curve of the relationship between *I*_Ho_/*I*_Er_ ratio and temperature.

Tissue sample	Slope
Skin + peritoneal muscle	0.026
Skin	0.024
Control	0.017

### *In Vitro* Characterization of a NaYF_4_ NP Silicone Composite

**Figure 5:**
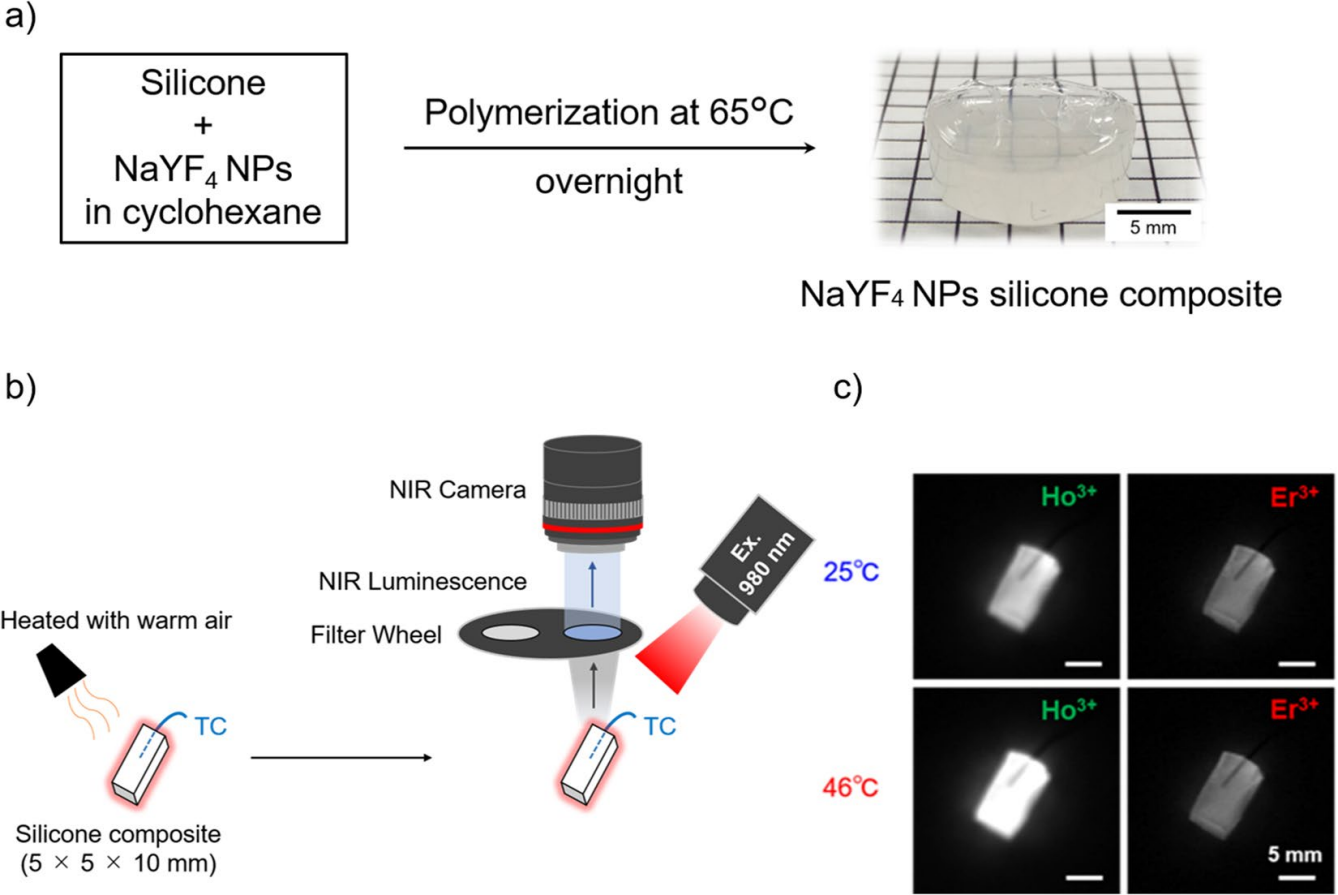
Fluorescence images of the silicone composite under 980-nm laser excitation (4 mW/cm^2^). (**a**) Preparation of the NaYF_4_ NP silicone composite. Silicone was mixed with NaYF_4_ NPs and polymerized overnight at 65 °C in a 24-well plate serving as a mold. Finally, the silicone composite containing the NaYF_4_ NPs (concentration; 20 mg/g) was obtained. (**b**) Schematic illustration of the imaging system for obtaining ratiometric fluorescence images using band-pass filters and an NIR camera (NIS-OPT). Ratiometric fluorescence images of the silicone composite were obtained via band-pass filters during the cooling process using an InGaAs camera (900–1700 nm spectral range). To acquire the fluorescence images at 1150 and 1550 nm quasi-simultaneously, two band-pass filters were placed on a motorized wheel. An image analysis software was used to calculate the ratio between the emission intensities at 1150 and 1550 nm. (**c**) The ratiometric fluorescence images of the silicone composite obtained by means of band-pass filters at 25 and 46 °C.

A composite of NaYF_4_ NP and silicone was prepared as shown in Fig. 5a, in order to evaluate the temperature dependence of the NaYF_4_ NPs emission while monitoring the temperature of NaYF_4_ NPs by insertion of thermocouple (TC) in deep region such as the peritoneal cavity, and to remove the effects of dispersion medium on LIR. The effectiveness of the silicone composite for application of LNTh to deep biological tissues was analyzed by acquiring the OTN-NIR emission spectra using a spectrometer and by capturing the fluorescence images using band-pass filters under 980-nm laser excitation in the physiological temperature rang (27–43 °C). Figure S2 (Supplementary Fig. S2) displays the emission spectra and the LIR curve of the silicone composite with temperature increase. The prepared composite shows emission bands of both Ho^3+^ at 1150 nm and Er^3+^ at 1550 nm under 980-nm laser excitation. In addition, Ho^3+^ emission of the composite depends on the change in temperature, while the Er^3+^ emission remains almost constant with temperature. Ho^3+^/Er^3+^ LIR value, therefore, is dependent on the temperature. As a result, the prepared composite functions like a nanothermometer that shows characteristic emission bands of Ho^3+^ and Er^3+^ under 980-nm excitation.

Next, the ratiometric fluorescence images of the prepared silicone composite were obtained using band-pass filters and NIR camera under 980-nm laser excitation. The temperature of the composite was recorded by a TC inserted into the silicone composite. The silicone composite was heated with warm air and introduced into the OTN-NIR fluorescence imaging system, as shown in Fig. 5b. The fluorescence images at 1150 and 1550 nm were captured at 25 and 46 °C during the relaxation process, as shown in Fig. 5c. The obtained images at these temperatures clearly reveal that the Ho^3+^ emission increases with temperature, whereas that of Er^3+^ remains almost constant with the change in temperature. Thus, fluorescence images with temperature change were obtained in this OTN-NIR fluorescence imaging system.

### Temperature Sensing in Deep Tissues by Using the NaYF_4_ NPs Silicone Composite

Having confirmed the temperature-dependent fluorescence intensity of the prepared composite by *in vitro* experiments, the ability of the composite to sense temperature changes in deep tissues was tested in deep tissues at the peritoneal cavity level. We investigated the possibility of acquiring emission spectra at the peritoneal cavity level. First, the temperature-dependent LIR change of the silicone composite was investigated via a simple experiment schematically described in Fig. S3a (Supplementary Fig. S3). The temperature-dependent emission spectra of the silicone composite at the peritoneal cavity level were recorded with a spectrometer under 980-nm laser excitation during the cooling dynamics of the tissue (Fig. S3 (Supplementary information)). As already demonstrated, the Ho^3+^ emission increased with an increase in temperature. In contrast, Er^3+^ emission remained almost constant. In addition, Fig. S3c (Supplementary Fig. S3) shows the temperature-dependent LIR change of the silicone composite at the peritoneal cavity level. These results confirm that the silicone composite shows temperature-dependent emission characteristics at the peritoneal cavity level.

**Figure 6:**
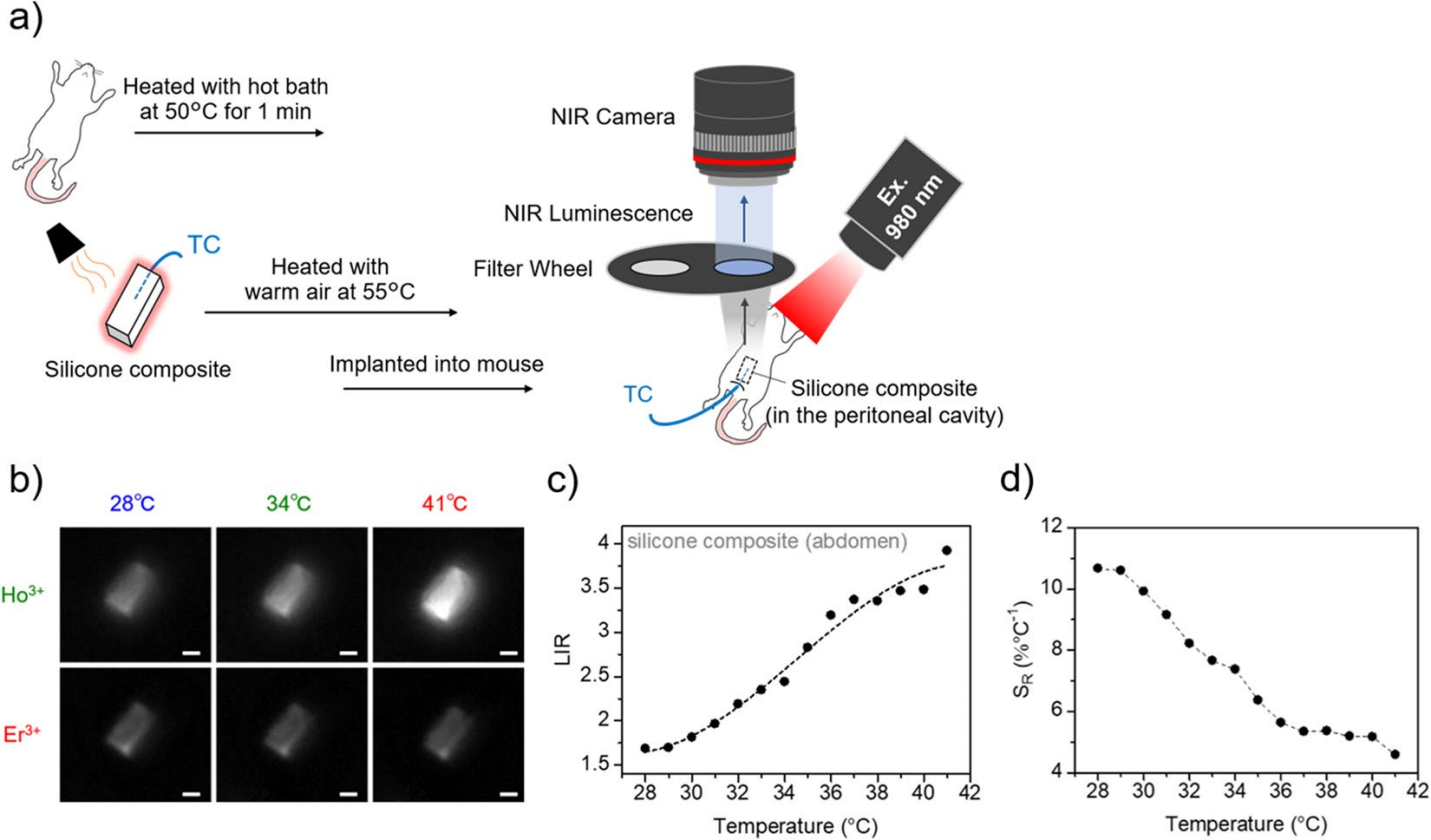
Temperature-dependent change in OTN-NIR fluorescence images of the silicone composite placed in the peritoneal cavity under 980-nm laser excitation. (**a**) Mouse was heated with a hot bath at 50 °C for 1 min and then the silicone composite was heated with warm air up to 55 °C and immediately implanted into the mouse at the peritoneal cavity level. Then, the ratiometric thermal images were acquired at different temperatures using band-pass filters at 1150 nm (Ho^3+^) and 1550 nm (Er^3+^) under 980-nm laser excitation (0.02 W/cm^2^) during the thermal relaxation process using OTN-NIR *in vivo* imaging system (NIS-OPT). (**b**) Ratiometric fluorescence images of the implanted area in the peritoneal cavity acquired using band-pass filters at 28, 34, and 41 °C. Scale bars represent 5 mm. (**c**) LIR calculated from each fluorescence image of the silicone composite located at the peritoneal cavity level. (**d**) The relative thermal sensitivity (*S*_R_) of the silicone composite containing NaYF_4_ NPs.

Then, the potential use of the silicone composite for thermal imaging was verified. As mentioned in the introduction, such thermal imaging at the peritoneal cavity level has not been performed before because of the low transparency of light in the VIS and NIR-I wavelength region^18,37,38^. Thus, the new imaging method at the deep tissue level suggested here would allow the development of *in vivo* thermal imaging in deeper tissues. The successful thermal imaging for deep tissues is demonstrated by our designed experiment schematically represented in Fig. 6a. The skin and peritoneal muscle of a dead mouse were cut and the silicone composite was implanted at the peritoneal cavity level. Because we had to insert the thermocouple into the deep peritoneal cavity to monitor the real temperature of the silicone composite in the deep area, we used a dead mice but a fresh one with no damage to skin or peritoneal muscle by preparing immediately after sacrifice by hyperanesthesia to qualify to claim *in vivo* thermal imaging. The body of dead mouse was warmed up with a hot bath at 50 °C for 1 min and positioned in the imaging system (NIS-OPT, Shimadzu Co., Kyoto, Japan). Then, the silicone composite inserted with the TC was immediately heated with warm air at 55 °C and implanted into the mouse. After fixing the position of the silicone composite inserted with the TC in the mouse body, we induced moderate thermal relaxation at the implanted site under 980-nm laser excitation. This experimental approach has been used to monitor the cooling dynamics of a tissue in previous studies of subcutaneous thermometry^31,32^. This heating approach, in which the body of the mouse is warmed up quickly and separately from the silicone composite suppresses heat damage to the mouse skin. Moreover, the composite in the mouse was excited by the 980-nm laser, with a low enough power density (0.02 W/cm^2^) to minimize additional heating, to record the fluorescence from the silicone composite placed in the peritoneal cavity. The temperature of the silicone composite was recorded by TC inserted in the peritoneal cavity.

The ratiometric fluorescence images of the implanted site were obtained using an InGaAs camera (900–1700 nm spectral range) during the thermal relaxation process. The gray value of the images was calculated and converted to the ratio between the fluorescence intensities of Ho^3+^ and Er^3+^ emissions at 1150 and 1550 nm, respectively, using an image analysis software. Figure 6b shows the fluorescence images obtained at 1150 and 1550 nm at 28, 34, and 41 °C, respectively. From these fluorescence images, the Ho^3+^ and Er^3+^ emissions from the silicone composite were detected from the deeper part, peritoneal cavity, using band-pass filters at both 1150 and 1550 nm. In addition, the Ho^3+^ emission intensity was dependent on the change in temperature, while the Er^3+^ fluorescence intensity remained almost constant. Figure 6c demonstrates the temperature dependence of the LIR between the Ho^3+^ and Er^3+^ emissions of the silicone composite at the peritoneal cavity level. It revealed that the LIR between the Ho^3+^ and Er^3+^ emissions calculated from each of the fluorescence images acquired at different temperatures increases with the temperature increase. The silicone composite containing NaYF_4_ NPs presents relative thermal sensitivities of 10.7 × 10^−2^ °C^−1^ and 4.59 × 10^−2^ °C^−1^, at 28 and 41 °C, respectively (Fig. 6d).

## Discussion

While the NaYF_4_ NPs emitted in the OTN-NIR range under 980-nm laser excitation, higher intensity Ho^3+^ emission was obtained in comparison with that of our previous report^33^ because we changed the dopant rare-earth concentration; previously Yb^3+^, Ho^3+^, and Er^3+^ concentrations of 20, 1, and 1 mol% were used, while in this work they were at 20, 3, and 0.5 mol%, respectively, as optimized according to a recently published work^34^. The maximum *S*_R_ of the optimized NPs is 1.87 × 10^−2^ °C^−1^. While other rare-earth-based LNThs operating in the OTN-NIR wavelength region (NIR-II and NIR-III) were reported recently^31,32,34,39–42^ (Table 2), we designed NaYF_4_ NPs only operating under 980-nm laser excitation in the OTN-NIR wavelength range. Indeed, most of the previously reported nanothermometers could be excited by 800-nm laser light, the absorption of which by water in biological tissues is relatively less than the absorption of the 980-nm laser light. Certainly, the use of 980-nm laser excitation might reduce the penetration depth because it is partially absorbed by the water within the tissues. The scattering of the 980-nm excitation light, however, is lower within the tissues during the OTN-NIR fluorescence bioimaging as compared to the 800-nm laser light. In case of irradiation with the 980-nm laser light from outside of the body noninvasively, the laser irradiation area would be minimized owing to low light scattering. In comparison with other works, our system has limitation; however, including other choices of excitation NIR wavelengths can be helpful for ensuing studies toward various imaging applications.

**Table 2:** Comparison of rare-earth based nanothermometers operating in the OTN-NIR (NIR-II and NIR-III) wavelength range.

Nanothermometers	Ex. (nm)	λ used for LIR (nm)	S_R_ (10^−2^ °C^−1^)	Observation depth	Reference
NaGdF_4_: Nd^3+^ & PbS/CdS/ZnS@PLGA	808	1060/1250	2.5	*In vitro*	^39^
YVO_4_: Nd^3+^	808	1063/1071	0.15	*In vitro*	^40^
T^NIR^-aqNPs^a^	806	1150/1330 1330/1550	1.16	*In vitro*	^41^
OA-NaYF_4_: Yb^3+^, Ho^3+^, Er^3+^	980	1150/1550	2.17	*In vitro*	^34^
LaF_3_: Nd^3+^, Yb^3+^	790	1000/1350	0.41	*In vivo*, subcutaneous	^31^
LaF_3_: Nd^3+^@Yb^3+^	808	1350/1000	0.74	*In vivo*, subcutaneous	^42^
LaF_3_: Er^3+^, Yb^3+^@Yb^3+^, Tm^3+^	690	1000/1230 1000/1550	5.0	*In vivo*, subcutaneous	^32^
NaYF_4_: Yb^3+^, Ho^3+^, Er^3+^	980	1150/1550	1.87	*In vivo*, peritoneal cavity	This work

^a^NaGdF_4_: Er^3+^, Ho^3+^, Yb^3+^@NaGdF_4_: Yb^3+^@NaGdF_4_: Nd^3+^, Yb^3+^@NaGdF_4_ coated with PEG-DOPE.

Abbreviations: Ex, excitation wavelength; S_R_, relative thermal sensitivity.

The present study shows for the first time that fluorescence NPs based on three rare-earth ions, Yb^3+^, Ho^3+^, and Er^3+^, could function as a nanothermometer for deeper tissues such as the peritoneal cavity under 980-nm laser irradiation. In the present work, we recorded the OTN-NIR emission spectra of LNTh through biological tissues for the first time and confirmed that the slope of the calibration curve is different corresponding to the thickness of the tissue. The differences in the slopes are due to the differences in the attenuation rates of Ho^3+^ and Er^3+^ emissions by the tissues (e.g., skin and skin + peritoneal muscle). The attenuation of the Er^3+^ emission by the tissues is larger than that of Ho^3+^ emission because the emission wavelength of Er^3+^ (~1550 nm) is close to the absorption wavelength of water within the tissues, 1440 nm. We hypothesized that the relative change of the LIR with temperature should not be affected by different transmittances through biological tissues at the measuring wavelengths; however, it is affected. The relative thermal sensitivity (S_R_) decreased as the thickness increased. This decrease is possibly due to shifting the absorption band of water in NIR to shorter wavelength by temperature increase^43,44^. This fact suggests that a calibration curve for temperature sensing would be required corresponding to the thickness. In the present study, a hydrophobic silicone composite was employed instead of a hydrophilic and biocompatible nanothermometer such as poly(ethylene glycol)-coated nanothermometer (e.g., poly(ethylene glycol)-modified NaYF_4_ NPs)^33^ to acquire fluorescence images in the peritoneal cavity. Temperature sensing in deeper parts using such hydrophilic and biocompatible nanothermometers would be required as a next step. Additionally, further experiments are required to better understand the mechanism of temperature-dependent change in LIR of the nanothermometer in deep tissue of mice. For example, the thermal relaxation time constant in the case of silicone composite covered with biological tissues will be an interest in next studies.

In conclusion, we first showed temperature-dependent changes in LIR between 1150 nm and 1550 nm emission of NaYF_4_ NPs placed in the peritoneal cavity of mice, which is deeper than the subcutaneous tissue, using fluorescence imaging technique in the NIR-II and NIR-III wavelength regions. The obtained LIR value changed corresponding to the thickness of the tissue because the optical loss by a biological tissue is dependent on the wavelength; however, the loss is relatively low in the OTN-NIR region. The relative change of LIR with temperature (*S*_R_) is also influenced by the depth, possibly due to the change in attenuation of fluorescence by water in the biological tissues by temperature increase. Therefore the relationship between the LIR or *S*_R_ and temperature should be calibrated by the depth of the nanothermometer *in vivo*. The LIR of the silicone composite showed temperature dependence and it successfully operated in the peritoneal cavity as a nanothermometer. The designed experimental system of temperature imaging in deeper regions as demonstrated here will open the pathway to novel LNTh at the peritoneal cavity level.

## Methods

### Synthesis of NaYF_4_: Yb^3+^, Ho^3+^, Er^3+^ NPs

NaYF_4_ NPs doped with Yb^3+^, Ho^3+^, and Er^3+^ ions were prepared by a thermal decomposition method reported elsewhere^45,46^ with minor modifications. Yttrium chloride hexahydrate (Sigma-Aldrich, St Louis, MO, USA) (0.78 mmol), ytterbium(III) chloride hexahydrate (Wako Pure Chemical Industries, Osaka, Japan) (0.20 mmol), holmium(III) chloride hexahydrate (Wako Pure Chemical Industries) (0.03 mmol), and erbium(III) chloride hexahydrate (Wako Pure Chemical Industries) (0.005 mmol) were mixed in a solvent composed of distilled water (0.67 mL), OA (14 mL; Sigma-Aldrich), and 1-octadecene (30 mL; Wako Pure Chemical Industries). After stirring at 160 °C for 60 min under a nitrogen atmosphere, the mixture was cooled to room temperature, and a solution of ammonium fluoride (NH_4_F, 4 mmol; Kanto Chemicals, Tokyo, Japan) and sodium hydroxide (NaOH, 2.5 mmol; Wako Pure Chemical Industries) in methanol (7 mL; Wako Pure Chemical Industries) was slowly added to the reaction mixture. After stirring, methanol in the mixture was removed by evaporation. Finally, the mixture was heated at 310 °C for 60 min under a nitrogen atmosphere. Finally, the NaYF_4_ NPs collected by precipitation were purified by centrifugal washing (20000 g, 10 min, × 3) with ethanol (Wako Pure Chemical Industries) and dispersed in cyclohexane (Wako Pure Chemical Industries).

### Structural Analysis of the NaYF_4_ NPs

The particle size distribution and morphology of the NaYF_4_ NPs were evaluated via TEM (HD-2300, Hitachi, Japan) with accelerating voltage of 200 kV. The crystalline phase of the NaYF_4_ NPs was evaluated via XRD (RINT-TTR III, Rigaku, Japan). The hydrodynamic diameters of the NaYF_4_ NPs samples (solvent: cyclohexane, concentration: 2 mg/mL, room temperature) were determined using a dynamic light scattering particle size analyzer (LB-550; Horiba, Ltd., Kyoto, Japan).

### Optical Characterization of the NaYF_4_ NPs

The temperature-dependent emission spectra of the NaYF_4_ NPs were recorded using a spectrometer (NIR-256-1.7, Avantes, Netherlands) equipped with a temperature-controlled cuvette holder (qpod 2e, Quantum Northwest, WA, USA) and a 980-nm excitation source, fiber-coupled laser diode (SP-976-5-1015-7, Laser Components, Ltd., Olching, Germany). Emission was collected through a 1050 nm long-pass filter placed between the sample cuvette (PSK-10, Sansyo Co., Ltd., Tokyo, Japan) and detector. For temperature measurements through actual biological tissues using the NaYF_4_ NPs, skin and peritoneal muscle were placed between the sample cuvette and the spectrometer. The biological tissues were placed between 2 glass slides. Before the measurement, physiological saline was dripped on the tissues to suppress drying.

### Preparation and Optical Characterization of the NaYF_4_ NPs Silicone Composite

A dispersion of NaYF_4_ NPs in cyclohexane were added to silicone. The NaYF_4_ NP-containing silicone was obtained by polymerization (NPs/silicone = 20 mg/g). The temperature-dependent emission spectra of the silicone composite were recorded using a spectrometer under 980-nm laser excitation. This laser fulfilled dual roles: it warmed up the silicone composite while simultaneously exciting the OTN-NIR-emitting ions of the NaYF_4_ NPs in the silicone matrix. The temperature variation was recorded by placing the TC in the silicone composite. The TC was inserted in the silicone composite in the vicinity of the area irradiated with 980-nm laser, without causing a sharp temperature increase under 980-nm laser excitation. For the ratiometric fluorescence imaging of the silicone composite at different temperatures, an InGaAs camera (XEVA-USB-FPA-320-100Hz, Xenics, Belgium) and band-pass filters (cut-on wavelength: 1175/50, 1550/30 nm) were employed. The temperature increase of the silicone composite was caused by the warm air.

### Animal Experiments

All the animal care and experiments were performed in accordance with the national and institutional guidelines for the care and use of laboratory animals with the approval of the Tokyo University of Science’s Animal Care and Use Committee. Four-week-old male ICR and BALB/c mice were purchased from SLC Inc. (Hamamatsu, Japan). After feeding with AIN-76A diet (Research Diets Inc., NJ, US) for >14 days to weaken the autofluorescence of the body, the mice were anesthetized, and their hair was removed to avoid light scattering. The temperature-dependent emission spectra of the silicone composite placed in the peritoneal cavity was collected using a spectrometer (NIR-256-1.7). Before the experiments, the animal was euthanized. First, the silicone composite was heated with a hot plate up to about ~60 °C. Second, the heated silicone composite was placed in the peritoneal cavity. Third, the OTN-NIR emission under 980-nm laser excitation was collected using the spectrometer. The temperature of the silicone composite was recorded using the TC. The ratiometric fluorescence images at different temperatures were obtained using an *in vivo* imaging system (NIS-OPT, Shimadzu Co., Kyoto, Japan) under 980-nm laser excitation. The NIR excitation light did not lead to additional heating of the silicone composite. The characteristic emission bands were obtained using the band-pass filter (cut-on wavelength: 1150/50 and 1550/50 nm). In some experiments, the mouse and silicone composite were warmed up to 50 °C using a hot bath and then the ratiometric images were obtained during thermal relaxation. The temperature of the silicone composite was registered by the inserted TC.

## Electronic supplementary material

Supporting Information

## Data Availability

The data required to reproduce these findings are available to download from https://data.mendeley.com/datasets/252vtt4dp5/draft?a=328737ac-4763-408c-a6b3-5590a4014f6f.
