# Supplementary material for: Temperature Sensing of Deep Abdominal Region in Mice by Using Over-1000 nm Near-Infrared Luminescence of Rare-Earth-Doped NaYF4 Nanothermometer
Source: Sci Rep. 2018 Nov 19;8:16979. doi: 10.1038/s41598-018-35354-y (PMC6242879; doi:10.1038/s41598-018-35354-y)
Supplement: Supplementary file 1 — Supporting Information [file 41598_2018_35354_MOESM1_ESM.docx]

**Supplementary Information**

**Temperature Sensing of Deep Abdominal Region in Mice by Using Over-1000 nm Near-Infrared Luminescence of Rare-Earth-Doped NaYF_4_ Nanothermometer**

Shota SEKIYAMA^1)^, Masakazu UMEZAWA^1,2,*)^, Shuhei KURAOKA^1)^, Takuji UBE^1)^, Masao KAMIMURA^1,2)^, Kohei SOGA^1,2,*)^

^1^Department of Materials Science and Technology, Faculty of Industrial Science and Technology, Tokyo University of Science, 6-3-1 Niijuku, Katsushika-ku, Tokyo 125-8585 Japan

^2^Imaging Frontier Center (IFC), Research Institute for Science and Technology (RIST), Tokyo University of Science, 2641 Yamazaki, Noda, Chiba 278-8510, Japan

(*Corresponding Authors)

**
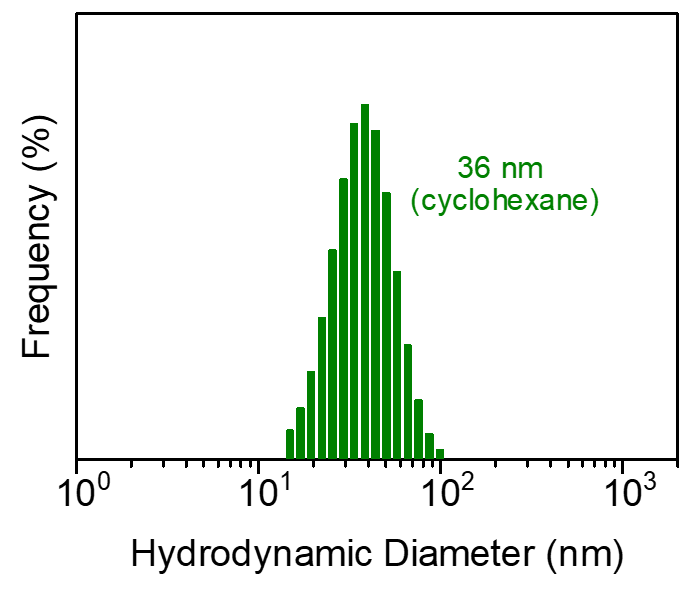
**

**Supplementary Figure S1.** Hydrodynamic diameter of the NaYF_4_: Yb^3+^, Ho^3+^, Er^3+^ NPs in cyclohexane determined using a dynamic light scattering particle size analyzer (LB-550; Horiba, Ltd.). The NPs concentration was 2 mg/mL.


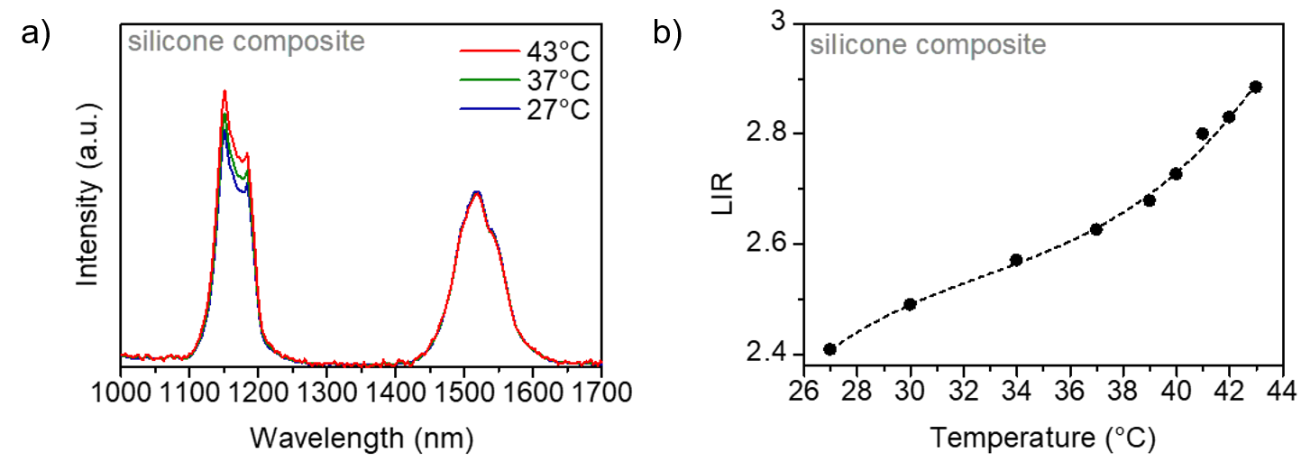


**Supplementary Figure S2.** Optical characterization of the silicone composite. a) OTN-NIR emission spectra of the silicone composite obtained at 27, 37, and 43°C under 980-nm laser excitation. b) Calibration curve of the silicone composite. The temperature-dependent emission spectra were recorded with a spectrometer under 980-nm laser excitation (4 mW/cm^2^). The temperature variation was recorded by placing the TC in the silicone composite.


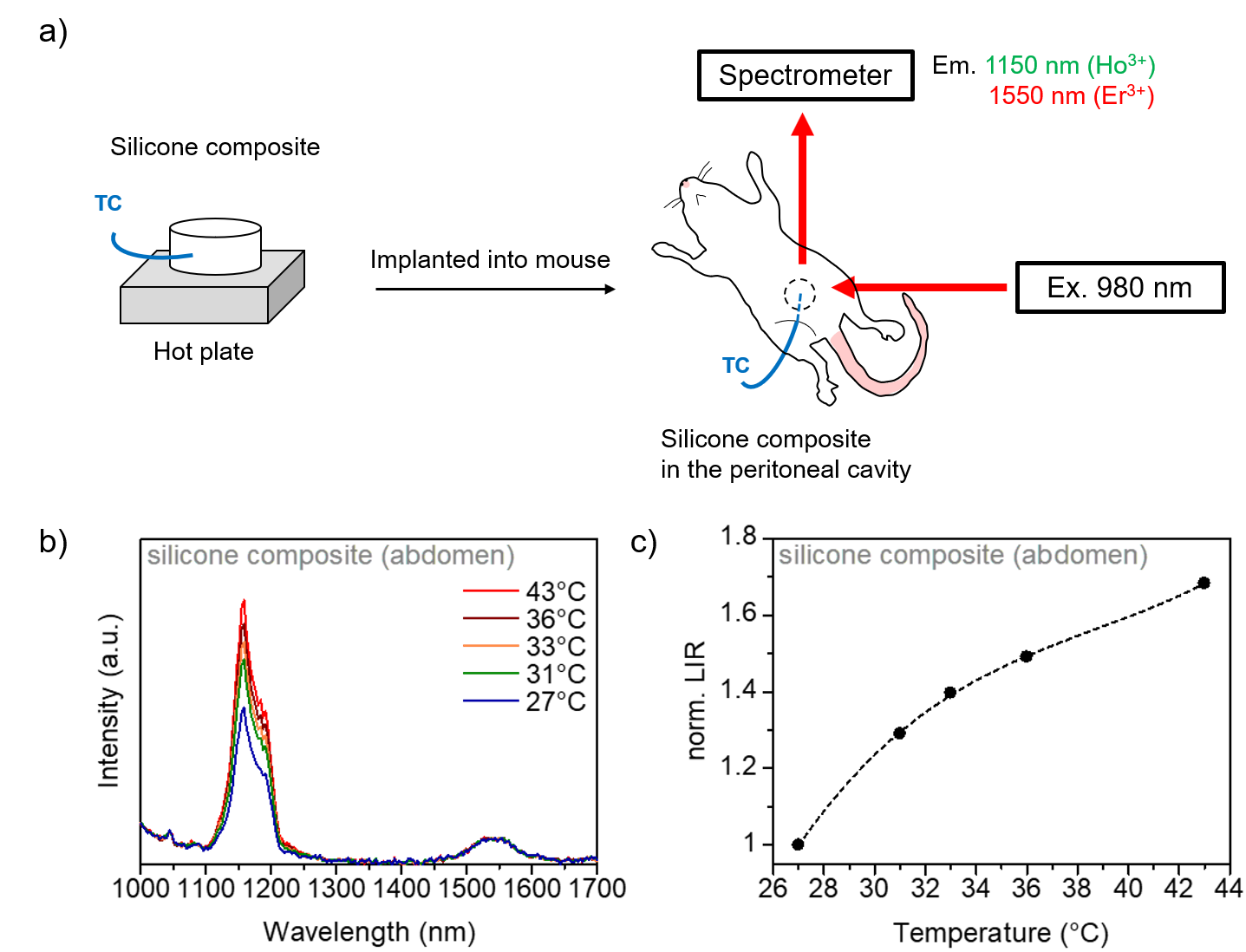


**Supplementary Figure S3.** Temperature-dependent change in OTN-NIR fluorescence spectra of the silicone composite placed in the peritoneal cavity. a) Schematic representing the acquisition of OTN-NIR fluorescence spectra of the silicone composite placed in mice. The silicone composite was heated with a hot plate and was placed in the peritoneal cavity of mice. b) OTN-NIR emission spectra of the silicone composite located at the peritoneal cavity level recorded at 27, 31, 33, 36, and 43°C under 980-nm laser excitation (0.02 W/cm^2^). c) Calibration curve of the silicone composite located at the peritoneal cavity level.
